# Evaluation of the Antioxidant, Antibacterial Activity and Volatile Components of Three Distinctive *Apis cerana* Honeys

**DOI:** 10.3390/foods15010024

**Published:** 2025-12-22

**Authors:** Haibo Wang, Cuiping Zhang, Caoyang Lu, Xiasen Jiang, Bin Yuan, Fuliang Hu, Defang Niu

**Affiliations:** 1 School of Food Science and Health, Jiangsu Agri-Animal Husbandry Vocational College, Taizhou 225300, China; jstzwhb@163.com (H.W.); 15195632682@163.com (C.L.); 2 College of Animal Sciences, Zhejiang University, Hangzhou 310058, China; lgzcplyx@zju.edu.cn (C.Z.); yuan_bin322@163.com (B.Y.); 3 Research Center of Anti-Aging Chinese Herbal Medicine of Anhui Province, Biology and Food Engineering School, Fuyang Normal University, Fuyang 236037, China; jiangxs@fynu.edu.cn

**Keywords:** *Apis cerana* honey, total phenolic content, antioxidant capacity, antibacterial activity, volatile compounds

## Abstract

To comprehensively evaluate the physicochemical characteristics and bioactivities of three distinctive *Apis cerana* (*A. cerana*) honeys—*Bauhinia championii* honey (BCH), *Polygonum perfoliatum* honey (PPH), and *Rhus chinensis* honey (RCH)—systematic analyses were performed on total phenolic content (TPC), in vitro antioxidant capacity (via DPPH, ABTS, and FRAP assays), antibacterial efficacy against *Staphylococcus aureus* (*S. aureus*) and *Escherichia coli*, and volatile composition analysis. Among the varieties, BCH presented the most substantial TPC (636.21 ± 17.05 mg GAE/kg), a value statistically higher than that of PPH and RCH (*p* < 0.05), and correspondingly exhibited superior antioxidant performance across all assays. All honeys displayed a clear concentration-dependent antioxidant effect. Antibacterial assays revealed that both undiluted and diluted (0.5 g/mL) samples exerted significant inhibitory effects on the tested pathogens, furthermore, BCH and PPH showing significantly higher inhibition against *S. aureus* than the positive control (penicillin, *p* < 0.05). Volatile profiling by HS-SPME-GC-QQQ identified 42, 35, and 38 volatile compounds in BCH, PPH, and RCH, respectively, including esters, terpenes, aldehydes, and phenolics. Cedrol (in PPH), phenylethyl acetate (in BCH), and benzaldehyde (in RCH) were pinpointed as potential botanical markers that define their characteristic aroma profiles and may partially contribute to their bioactivity. Collectively, these honeys possess remarkable antioxidant and antibacterial properties, with BCH showing the greatest potential for functional food applications owing to its high phenolic concentration and robust overall bioactivity. These findings elucidate the intrinsic link between the botanical chemical signatures of *A. cerana* honey and its biological functions, offering a scientific foundation for their utilization in functional food innovation, geographical indication protection, and quality authentication.

## 1. Introduction

The convergence of honey’s role as a natural sweetener and its status as a traditional food-medicine homologous product has placed it at the forefront of contemporary food science and nutritional research. Its growing prominence is attributed to a remarkable nutritional value and diverse biological functionalities [1,2]. The bioactive potential of honey, which includes antioxidant, antimicrobial, anti-inflammatory, and immunomodulatory properties, is largely conferred by a complex matrix of non-carbohydrate components such as polyphenols, flavonoids, enzymes, and organic acids [3,4,5,6]. As consumer demand for natural, healthy, and functional foods continues to rise, the scientific exploration and detailed characterization of honeys from specific botanical origins have become increasingly crucial. Such efforts are vital for driving the high-quality development and functional enhancement of the honey industry.

The botanical origin of honey fundamentally dictates its chemical composition, sensory attributes, and biological activities [7]. Consequently, a systematic examination of monofloral honeys is crucial not only for elucidating their distinct functional and chemical profiles but also for establishing their precise market value. In China, several unique honeys produced by *Apis cerana* (*A. cerana*) are sourced from medicinal plants, possessing significant ethnopharmacological relevance and regional specificity, which endows them with considerable scientific and economic importance [8]. This study investigates three representative varieties: (1) *Bauhinia championii* honey (BCH), derived from *Bauhinia championii* (*Benth.*) *Benth* (Fabaceae), a plant whose extracts are documented for their anti-inflammatory, antioxidant, and immunomodulatory effects [9]; (2) *Polygonum perfoliatum* honey (PPH), produced from *Polygonum perfoliatum* L. (Polygonaceae), a traditional medicinal herb recognized for its heat-clearing, detoxifying, and anti-swelling properties [10]; and (3) *Rhus chinensis* honey (RCH), collected from *Rhus chinensis Mill.* (Anacardiaceae), whose fruits and galls are abundant in tannins and phenolics and are broadly applied for their astringent, antibacterial, and antioxidant applications [11]. Given the pharmacological characteristics of these nectar plants, their corresponding honeys are expected to contain unique bioactive compounds with potential for developing novel natural antioxidants and functional food ingredients.

However, comprehensive studies on these three honey varieties are still insufficient. To date, research has primarily addressed their fundamental physicochemical properties, lacking systematic comparisons of key quality indicators like total phenolic content and biological activities, including antioxidant and antibacterial performance. As a result, the scientific evidence needed to support their nutritional claims and market recognition is inadequate. Prior studies have shown that the total phenolic content does not fully account for the antioxidant activity of honey. Discrepancies between phenolic concentration and antioxidant assay results may indicate the involvement of other bioactive compounds, such as certain volatile constituents, which act synergistically to produce antioxidant effects [12,13]. Nonetheless, this hypothesis has not yet been thoroughly investigated.

The volatile profile of honey, which dictates its unique aroma, also provides a chemical signature for authenticating its botanical and geographical sources [14,15]. Leveraging these characteristic markers to build discrimination models is critical for protecting geographical indications, preventing fraud, and standardizing production and quality protocols.

This study was conducted to provide a comprehensive physicochemical and functional characterization of BCH, PPH, and RCH. The specific objectives were to: (1) determine and compare their total phenolic content and in vitro antioxidant potential using DPPH, ABTS, and FRAP assays; (2) assess their antibacterial efficacy against *Staphylococcus aureus* (*S. aureus*) and *Escherichia coli* (*E. coli*); (3) map their volatile profiles via gas chromatography–triple quadrupole mass spectrometry (HS-SPME-GC-QQQ) to establish key markers for botanical origin and quality authentication; and (4) establish correlations between the identified active compounds and the measured bioactivities to uncover potential “dual-functional” constituents.

This research provides a comprehensive functional and aromatic profile of three distinct *A. cerana* honeys through an integrated analysis of their chemistry and bioactivity. The resulting insights offer a dual scientific contribution: a practical basis for botanical authentication, quality standardization, and functional food innovation, coupled with a theoretical framework to support brand development and high-value utilization in the Chinese honey industry.

## 2. Materials and Methods

### 2.1. Samples of Honey

Three monofloral *A. cerana* honey samples—BCH, PPH, and RCH—were procured from local beekeepers. Their botanical origins are summarized in Table 1. Each honey type (BCH, PPH, and RCH) consisted of three independent batches collected from local beekeepers, and the three batches of each honey type were pooled to obtain a representative composite sample for analysis. The samples were then kept at −4 °C in darkness until analysis.

To verify the botanical origin of the three honey samples, a melissopalynological analysis was performed using the method outlined by Belay et al. [16], with some minor adjustments. The predominant pollen types corresponding to the declared floral sources accounted for between 75.35% and 83.40% of the total pollen grains, exceeding the commonly accepted threshold for monofloral honey. These results confirm that BCH, PPH and RCH were all high-purity monofloral honeys.

### 2.2. Experimental Materials

All reagents were of analytical grade or higher. The bacterial strains, *S. aureus* (ATCC 29213) and *E. coli* (ATCC 25922) were obtained from Shanghai Beinuo Biotechnology Co., Ltd. (Shanghai, China). Antioxidant assay standards, including 1,1-diphenyl-2-picrylhydrazyl (DPPH), 2,2′-azino-bis(3-ethylbenzothiazoline-6-sulfonic acid) diammonium salt (ABTS), and gallic acid, along with Folin reagent, were sourced from Merck, Shanghai Aladdin (Shanghai, China), and Shanghai Beyotime Biotechnology (Shanghai, China) companies, respectively. The total antioxidant capacity assay kit (FRAP method) was also purchased from Shanghai Beyotime Biotechnology Co., Ltd. Chromatographic-grade solvents (acetonitrile and methanol) and other analytical-grade chemicals (potassium persulfate, sodium carbonate, anhydrous ethanol) were obtained from Merck and Sinopharm Chemical Reagent Co., Ltd. (Shanghai, China), respectively. Nutrient broth and agar for microbiological culture were supplied by Haibo Biotechnology Co., Ltd. (Taizhou, China).

### 2.3. Experimental Procedure

#### 2.3.1. Quantification of Total Phenolics

The total phenolic content in honey samples was quantified via the Folin–Ciocalteu colorimetric assay, a method adapted from Wilczyńska et al. [17]. A calibration curve was established using gallic acid standards (0.04–0.28 mg/mL), which yielded the linear regression equation y = 1.1961x + 0.0256 (R^2^ = 0.9994). The analytical procedure was as follows: 10 μL of a honey solution (0.2 g/mL) was combined with 50 μL of Folin–Ciocalteu reagent (0.2 mol/L). After a 5 min reaction, 40 μL of sodium carbonate solution (0.075 g/mL) and 100 μL of distilled water were sequentially added. The resulting mixture was incubated for 2 h at room temperature in the dark, after which its absorbance was recorded at 760 nm. The final results were expressed as milligrams of gallic acid equivalents per 100 g of honey (mg GAE/100 g).

#### 2.3.2. Evaluation of Antioxidant Activity

##### DPPH Radical Scavenging Assay

The DPPH radical scavenging activity of honey was evaluated according to the procedures described by Almeida-Muradian et al. [18], with some modifications. A 0.2 mmol/L DPPH working solution was initially prepared in anhydrous ethanol, which was stored at 4 °C in the dark and freshly prepared prior to each assay. Honey samples were diluted to a series of concentrations (0.05–0.30 g/mL). For the analysis, 100 μL of each diluted honey sample was pipetted into a well of a 96-well microplate and mixed with 100 μL of the DPPH solution. The mixture was then incubated for 30 min at room temperature, protected from light, and its absorbance was subsequently measured at 517 nm. A standard curve was generated using vitamin C (ascorbic acid) at concentrations of 1–10 μg/mL, which followed the regression equation y = −0.0649x + 0.8148 (R^2^ = 0.9993). The antioxidant capacity was ultimately expressed as milligrams of ascorbic acid equivalents per 100 g of honey (mg AAE/100 g).

##### ABTS Radical Scavenging Assay

The ABTS radical cation decolorization assay was conducted based on the protocols of Almeida-Muradian et al. [18], with modifications. To generate the ABTS stock solution, 5 mL of a 7 mmol/L ABTS solution was reacted with 89 μL of a 140 mmol/L potassium persulfate solution. The mixture was left to stand in the dark at room temperature for 16 h to ensure complete radical generation. Prior to analysis, this stock solution was diluted with ethanol to produce a working solution with an absorbance of 0.700 ± 0.002 at 734 nm. Honey samples were prepared at concentrations ranging from 0.10 to 1.00 g/mL. For the assay, 100 μL of each honey solution was combined with 100 μL of the ABTS working solution in a 96-well microplate. After a 6 min incubation at room temperature in the dark, the absorbance was measured at 734 nm. A calibration curve was established using Trolox standards (0.002–0.025 mmol/L), which yielded the regression equation y = −11.707x + 0.3359 (R^2^ = 0.9993). The antioxidant capacity was expressed as millimoles of Trolox equivalents (TE) per kilogram of honey (mmol TE/kg).

##### FRAP Reducing Power Assay

The Ferric Reducing Antioxidant Power (FRAP) of honey was assessed using a commercial Total Antioxidant Capacity Assay Kit, which operates on the FRAP principle. For quantification, a calibration curve was established with FeSO_4_ standards over a concentration range of 0.15–1.50 mmol/L, resulting in the regression equation y = 0.6721x + 0.0498 (R^2^ = 0.9991). Honey samples were diluted to prepare a series of concentrations from 0.05 to 0.40 g/mL. The assay was performed by mixing 20 μL of each honey solution with 180 μL of freshly prepared FRAP working solution. The reaction mixture was then incubated at 37 °C for 5 min, after which the absorbance was recorded at 593 nm. The final results were expressed as millimoles of FeSO_4_ equivalents (FE) per gram of honey (mmol FE/g).

##### EC50 Calculation

The half-maximal effective concentration (EC_50_) is the concentration of a sample required to achieve a 50% scavenging of free radicals in a defined system. It is a key parameter for evaluating the potency of an antioxidant. A lower EC_50_ value indicates higher antioxidant activity, as it signifies that less of the sample is needed to elicit the same effect. This metric is widely used in radical scavenging assays, such as the DPPH and ABTS methods.

The radical scavenging rate was calculated using the following equation:

Scavenging Rate (%) = [(A_0_ − As)/A_0_] × 100% (1)

where

A_0_ is the absorbance of the blank control (sample-free).
As is the absorbance of the sample solution containing honey at a specific mass concentration.

To determine the EC_50_, plot the honey mass concentration on the x-axis against the corresponding scavenging rate on the y-axis. A regression equation is then fitted to the data points. The EC_50_ value is the concentration corresponding to a 50% scavenging rate, which can be calculated from this regression equation.

#### 2.3.3. Evaluation of Antibacterial Activity

The antibacterial activity of honey against *S. aureus* and *E. coli* was investigated using a modified Oxford cup assay [19]. Initially, the bacterial strains were activated by inoculation into nutrient broth and incubating at 37 °C for 24 h. Subsequently, the activated cultures were transferred to fresh sterile nutrient broth and incubated with shaking at 37 °C to reach the stationary phase (12 h for *E. coli* and 48 h for *S. aureus*). The final bacterial suspensions were adjusted to a concentration of approximately 1 × 10^7^ colony-forming units (CFU)/mL for use in the assay, as determined via the plate colony counting method.

To prepare the assay plates, nutrient agar was dissolved in ultrapure water, sterilized at 121 °C for 15 min, and cooled to approximately 50 °C. The molten agar was then inoculated with the bacterial suspension at a 1:100 (*v*/*v*) ratio, mixed thoroughly, and approximately 20 mL was poured into sterile Petri dishes to solidify. Prior to loading into the Oxford cups, all honey samples were examined to confirm their physical state. Each honey was in a fully liquid, non-crystallized state at the time of analysis. For undiluted samples, honey was gently warmed in a 37 °C water bath for 10 min to ensure homogeneity without altering its chemical composition. Four sterile Oxford cups were placed on the surface of each solidified agar plate. Into each cup, 100 μL of a different test solution was added: a 50% (*w*/*v*) honey solution (0.5 g/mL), undiluted honey, a positive control (a mixture of penicillin, 10,000 U/mL, and streptomycin, 4000 U/mL), and a negative control (sterile distilled water).

All antibacterial assays were conducted using three independent biological replicates, and each biological replicate was measured in triplicate technical replicates to ensure reproducibility. The plates were incubated at 37 °C for 16 h (*S. aureus*) or 12 h (*E. coli*). Following incubation, the diameters of the resulting inhibition zones were measured in millimeters using a digital caliper.

#### 2.3.4. Analysis of Volatile Components

##### HS-SPME Extraction

The volatile constituents of the honey samples (detailed in Table 1) were extracted using headspace solid-phase microextraction (HS-SPME). To ensure efficient adsorption, a 50/30 μm DVB/CAR/PDMS fiber (Supelco 57328-U, Supelco Inc., Darmstadt, Germany) was utilized. For each extraction, approximately 1.5 g of honey was weighed into an 8 mL headspace vial, to which 0.3 mL of deionized water and a magnetic stir bar were added to facilitate homogenization. The sealed vial was placed in a water bath at 70 °C for a 10 min equilibration period. Subsequently, the SPME fiber was exposed to the headspace for 50 min to adsorb the volatiles. Following extraction, the fiber was immediately transferred to the GC-MS injection port for thermal desorption at 230 °C for 5 min. Prior to each use, the fiber was reconditioned at 250 °C for 5 min to prevent carryover.

##### GC-MS Instrumental Parameters

Chromatographic and mass spectrometric analyses were carried out on an Agilent 7890B-7000C GC-MS system (Agilent Technologies, Inc., Santa Clara, CA, USA) equipped with a 30 m × 0.25 mm × 0.25 µm capillary column. The injection was performed in splitless mode with the injector temperature maintained at 250 °C. Helium served as the carrier gas at a constant flow rate of 1.0 mL/min. The column oven temperature was programmed as follows: initially held at 40 °C, then ramped to 160 °C at a rate of 4 °C/min (held for 3 min), and subsequently increased to 280 °C at 10 °C/min (held for 3 min). The mass spectrometer was operated with the detector temperature set to 250 °C, and mass spectra were acquired across a mass-to-charge (*m*/*z*) range of 30–550.

##### Compound Identification

Identification of volatile compounds was achieved by a dual approach: comparing their experimental mass spectra with reference spectra from the National Institute of Standards and Technology (NIST 17) library, and by calculating their retention indices (RIs) against a C7–C40 n-alkane standard mixture. A compound was considered positively identified when its mass spectral similarity exceeded 80% [20]. No internal standard was used for quantification. Volatile compounds were semi-quantified based on their relative peak areas obtained from total ion chromatograms (TIC), expressed as percentage contributions to total volatile content.

### 2.4. Statistical Analysis

All measurements were performed in triplicate, and the data are presented as mean ± standard deviation (SD). Data processing and graphical visualization were conducted using Microsoft Excel 365. Statistical significance among the different honey samples was determined using one-way analysis of variance (ANOVA) in SPSS 26.0 software, followed by Duncan’s multiple range test for post hoc comparisons. A *p*-value of less than 0.05 (*p* < 0.05) was considered to indicate statistical significance.

## 3. Results and Discussion

### 3.1. Total Polyphenols of Honey

The TPC of three A. cerana honeys from different origins—BCH, PPH, and RCH—were quantified as 636.21 ± 17.05, 534.39 ± 106.47, and 496.72 ± 42.92 mg GAE/kg, respectively. BCH demonstrated a significantly higher TPC compared to PPH and RCH (*p* < 0.05), while the difference between PPH and RCH was not statistically significant (*p* > 0.05). These values were generally higher than those reported for other honeys, including buckwheat (*Fagopyrum esculentum*), chestnut (*Castanea sativa*), citrus (*Citrus*), cotton (*Gossypium hirsutum*), Jerusalem thorn (*Paliurus spina-christi*), pine (*Pinus*), oak (*Quercus*), thyme (*Thymus capitatus*), acacia honey (*Robinia pseudoacacia*), linden honey (*Tilia* spp.) and rapeseed honey (*Brassica napus*), which ranged from 72.1 ± 45.7 mg GAE/100 g to 274.467 ± 6150 mg GAE/kg [21,22,23,24,25]. Their TPCs also surpassed that of *Apis mellifera* honey from the same botanical sources (BCH: 406.29 ± 18.48 mg GAE/kg; RCH: 164.90 ± 20.37 mg GAE/kg) [25]. Nevertheless, their TPCs were lower than those of manuka (*Leptospermum scoparium*) fennel (*Foeniculum vulgare*) and *Vernonia amygdalina* honey, which are renowned for their exceptionally high phenolic levels ranging from 693.3 ± 26.8 to 862.28 ± 27.01 mg GAE/kg [26,27]. These findings suggest that honey’s phenolic content is not uniform, but instead varies according to botanical origin, bee species and environmental factors such as harvest time.

### 3.2. Antioxidant Capacity

The antioxidant activities of BCH, PPH and RCH were systematically evaluated using DPPH, ABTS and FRAP assays. These results were then expressed as standard equivalents (AAE, TE and FE) to provide a robust quantitative measure across concentrations.

#### 3.2.1. DPPH Radical Scavenging Activity

**Figure 1:**
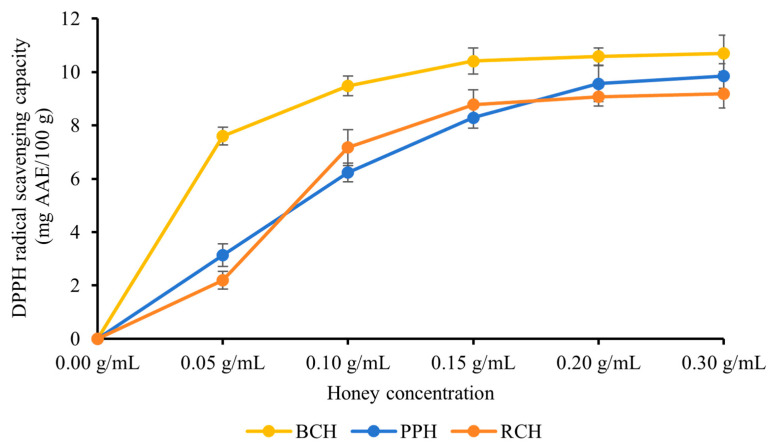
DPPH radical scavenging capacity of BCH, PPH, and RCH honey. Results are expressed as mg of ascorbic acid equivalents (AAE) per 100 g of honey. Data are presented as mean ± standard deviation.

All three honey varieties—BCH, PPH and RCH—exhibited potent scavenging activity against DPPH free radicals. As illustrated in Figure 1, a clear concentration-dependent relationship was observed within the tested range of 0.05–0.30 g/mL, where the scavenging capacity progressively increased with higher honey concentrations. At the peak concentration of 0.30 g/mL, the scavenging activities reached their maximum values, recorded as (10.69 ± 0.68) mg AAE/100 g for BCH, (9.85 ± 0.46) mg AAE/100 g for PPH, and (9.17 ± 0.54) mg AAE/100 g for RCH. Statistical analysis revealed that BCH’s DPPH radical scavenging capacity was significantly superior to that of both PPH and RCH (*p* < 0.05). This gradient is consistent with the TPC data, supporting the notion that phenolic compounds are primary contributors to DPPH radical scavenging [28,29]. The stronger activity of BCH likely reflects both higher phenolic concentration and potentially more effective phenolic composition.

As shown in Table 2, the three honey samples exhibited distinct DPPH radical scavenging activities. BCH demonstrated the most potent activity, with a significantly lower EC_50_ value of (0.015 ± 0.002) g/mL compared to both PPH (0.185 ± 0.012) g/mL and RCH (0.207 ± 0.018) g/mL (*p* < 0.05). In contrast, the difference between PPH and RCH was not statistically significant (*p* > 0.05). These results indicate that the antioxidant capacity of the honeys ranked as BCH > PPH ≈ RCH, with BCH being the most effective scavenger, followed by PPH and RCH which showed comparable activities.

#### 3.2.2. ABTS Radical Scavenging Activity

**Figure 2:**
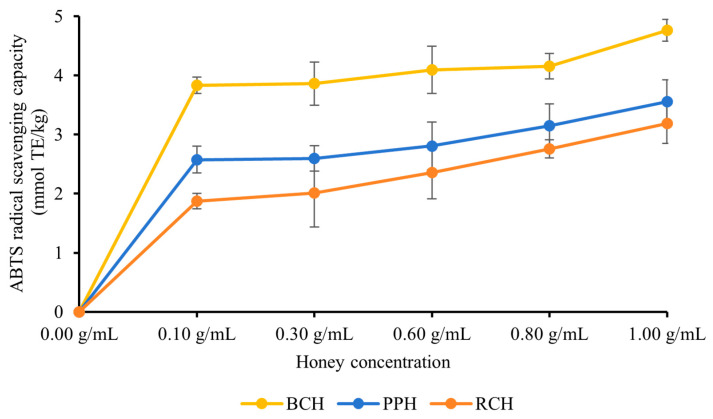
ABTS radical scavenging capacity of three types of honey. The results are expressed in mmol TE/kg (Trolox equivalent, TE). Data are presented as mean ± standard deviation.

Similar to the DPPH assay, all three *A. cerana* honey varieties (BCH, PPH, and RCH) demonstrated potent scavenging capabilities against ABTS radicals. As depicted in Figure 2, a clear concentration-dependent enhancement in the ABTS radical scavenging rate was observed across the 0.10–1.00 g/mL range, signifying an increase in antioxidant potential. At each tested concentration, significant differences were noted among the honeys (*p* < 0.05), with the scavenging potency consistently following the hierarchy of BCH > PPH > RCH. The peak activities were recorded at the maximum concentration of 1.00 g/mL, with values of (4.77 ± 0.18) mmol TE/kg for BCH, (3.56 ± 0.36) mmol TE/kg for PPH, and (3.19 ± 0.34) mmol TE/kg for RCH. The significant differences among honeys (*p* < 0.05) and the consistency with DPPH results suggest that BCH has a higher capacity for scavenging radicals, which is probably due to the quantity and quality of its phenolic constituents.

As shown in Table 3, the three honey samples demonstrated varying ABTS radical scavenging activities. Based on the EC_50_ values, PPH exhibited the most potent scavenging ability, with the lowest EC_50_ of (0.689 ± 0.018) g/mL. This was followed by RCH, with an EC_50_ of (0.720 ± 0.078) g/mL. In contrast, BCH showed the weakest activity, possessing the highest EC_50_ of (0.880 ± 0.012) g/mL. Statistical analysis of the 95% confidence intervals revealed that the antioxidant capacity of PPH was significantly higher than that of BCH (*p* < 0.05). However, no significant differences were found between PPH and RCH, or between RCH and BCH.

#### 3.2.3. FRAP Assay

**Figure 3:**
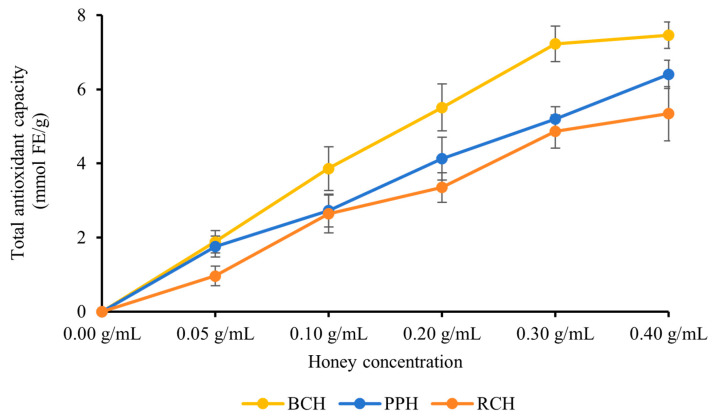
FRAP of the three honeys. Results are expressed as mmol of ferrous sulfate equivalents (FeSO_4_/g). Data are presented as mean ± standard deviation.

The FRAP of the three *A. cerana* honey varieties (BCH, PPH, and RCH) was assessed using the FRAP assay over a concentration gradient of 0.05–0.40 g/mL. As shown in Figure 3, all honey samples displayed potent reducing power that intensified in a concentration-dependent manner. A general trend of BCH > PPH > RCH was observed for total antioxidant capacity across most concentrations, with these differences being statistically significant (*p* < 0.05). The sole exception to this pattern occurred at 0.30 g/mL, where the difference between PPH and RCH was not statistically significant (*p* > 0.05). At the peak concentration of 0.40 g/mL, the FRAP values reached their maximum, recorded as (9.46 ± 0.36) mmol FE/g for BCH, (6.40 ± 0.38) mmol FE/g for PPH, and (5.34 ± 0.73) mmol FE/g for RCH, with significant differences among all three (*p* < 0.05). The minor variations observed between PPH and RCH at certain concentrations suggest that the presence of other bioactive components, such as flavonoids, vitamins or enzymes, may result in a synergistic effect.

In summary, the findings from this study demonstrate that the antioxidant capacities of the three *A. cerana* honey varieties (BCH, PPH, and RCH) are significantly enhanced in a concentration-dependent manner. A consistent hierarchy of antioxidant potential was established, with BCH exhibiting markedly superior activity compared to both PPH and RCH (*p* < 0.05), following the order of BCH > PPH > RCH. Although the performance gap between PPH and RCH narrowed at elevated concentrations, the preeminence of BCH’s antioxidant activity remained unequivocal. When integrated with the TPC data, a strong positive correlation was observed between in vitro antioxidant capacity and total phenolic levels, suggesting that phenolic compounds are the principal contributors to the honey’s antioxidant effects. Nevertheless, it is acknowledged that other bioactive constituents, such as flavonoids, vitamins, and enzymes, may also play a synergistic role [30]. The specific contributions of these components warrant further comprehensive investigation. It is important to note that these antioxidant results are derived solely from in vitro assays and cannot be directly extrapolated to in vivo effects. The antioxidant potential of honeys in chemical terms does not necessarily reflect their physiological activity, which depends on bioavailability and metabolism. Further in vivo validation is required.

### 3.3. Antibacterial Activitys

**Figure 4:**
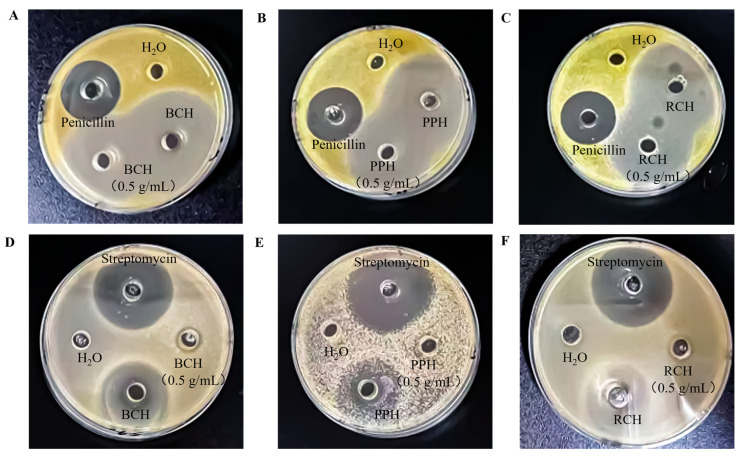
Antibacterial activity of *A. cerana* honeys against *S. aureus* and *E. coli* as demonstrated by inhibition zone assays. (**A**–**C**) Inhibition zones produced by undiluted and 0.5 g/mL solutions of BCH, PPH, and RCH against *S. aureus*. (**D**–**F**) Corresponding inhibition zones against *E. coli*. For each assay, sterile distilled water was used as the negative control, with penicillin and streptomycin serving as the respective positive controls.

The antibacterial efficacy of the honey samples against *S. aureus* and *E. coli* was assessed by measuring inhibition zone diameters (Table 4, Figure 4). Against the Gram-positive bacterium *S. aureus*, all honey samples (BCH, PPH, RCH), in both undiluted and 0.5 g/mL forms, demonstrated significantly greater antibacterial activity than the penicillin positive control (*p* < 0.05), corroborating previous reports [28,29]. For the undiluted samples, the inhibition zones were (50.21 ± 0.40) mm for BCH, (49.36 ± 0.80) mm for PPH, and (47.15 ± 0.56) mm for RCH. These results reveal that BCH and PPH possess superior inhibitory effects against *S. aureus* compared to RCH.

In contrast, against the Gram-negative bacterium *E. coli*, while the honeys still exhibited notable activity, their efficacy was significantly lower than that of the streptomycin positive control (*p* < 0.05). A clear concentration effect was observed, as undiluted honeys produced significantly larger inhibition zones than their 0.5 g/mL counterparts (*p* < 0.05). The inhibitory potency against *E. coli* followed a distinct ranking of PPH > RCH > BCH, with corresponding inhibition zone diameters of (30.15 ± 0.42) mm, (27.18 ± 0.50) mm, and (18.23 ± 0.14) mm for the undiluted samples.

### 3.4. Volatile Components Analysis

#### 3.4.1. Profiling and Comparison of Volatile Compounds

**Figure 5:**
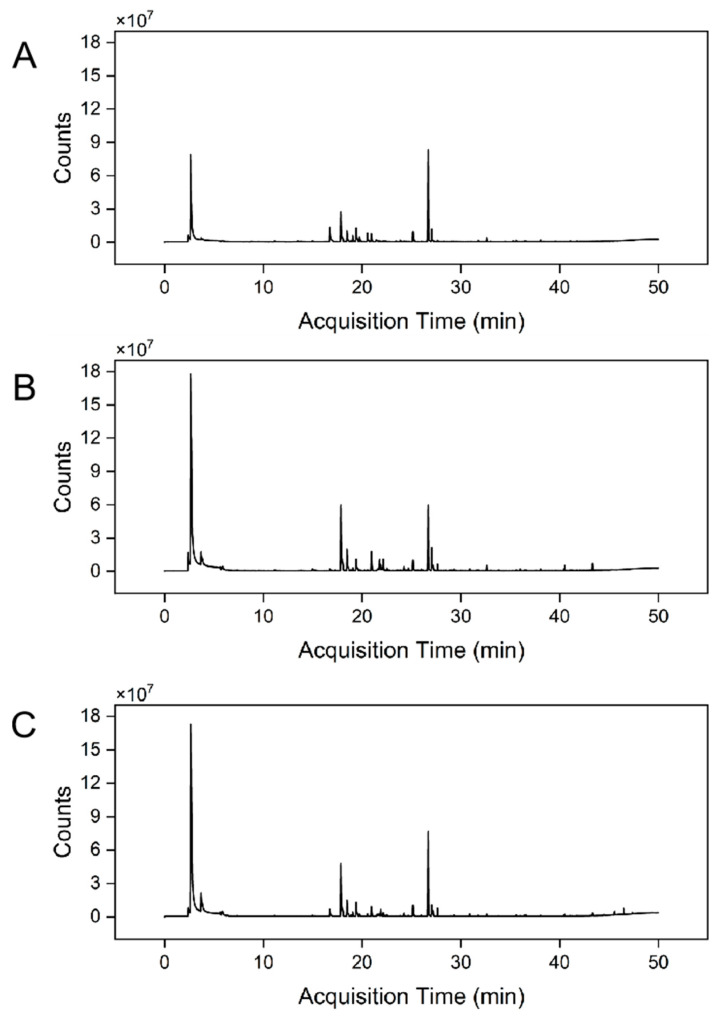
TICs of volatile components in three honey varieties. (**A**) BCH Sample, (**B**) PPH Sample, (**C**) RCH Sample.

The volatile profiles of the three *A. cerana* honey varieties (BCH, PPH, and RCH) were comprehensively characterized using headspace solid-phase microextraction coupled with HS-SPME-GC-QQQ. The resulting total ion chromatograms (TICs) are presented in Figure 5. Through mass spectral matching against the NIST library and retention index (RI) verification, a total of 41, 35, and 38 distinct volatile compounds were successfully identified in BCH, PPH, and RCH, respectively. These identified constituents were primarily classified into several chemical families, including esters, alcohols, aldehydes, terpenes and their derivatives, heterocyclic compounds, and phenols, as detailed in Tables S1–S3.

**Figure 6:**
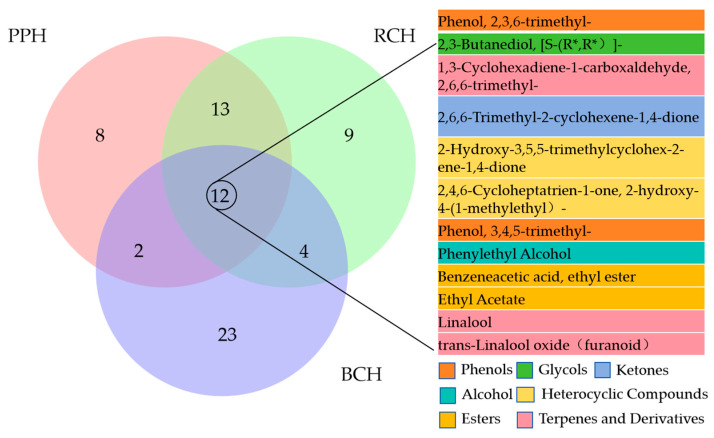
Venn diagram showing the volatile components found in three honey samples. The core compounds shared by all three honeys are listed and highlighted on the right-hand side of the diagram.

A core set of 12 volatile compounds was common to all three honey types, establishing a fundamental aromatic foundation (Figure 6). In contrast, each variety possessed a unique profile of characteristic volatiles, with BCH, PPH, and RCH containing 23, 8, and 9 exclusive compounds, respectively. The shared volatile profile was predominantly shaped by esters (e.g., ethyl acetate, phenethyl acetate), which were the most abundant class and imparted key floral and fruity notes. This core aroma was further enriched by alcohols like phenethyl alcohol and 2,3-butanediol, which added floral and fruity nuances while also contributing antioxidant properties. Aldehydes (e.g., 2,6,6-trimethyl-1,3-cyclohexadiene-1-carboxaldehyde) provided characteristic almond and floral scents and are recognized for their bioactivity, including antibacterial and antioxidant effects. Ketones contributed floral or fruity scents, and some may also possess biological activity. Terpenes and their derivatives are key components of natural fragrances, imparting complex woody and floral notes to honey. Phenolic compounds likely originated from pollen, plant metabolites, or fermentation products, contributing unique aromatic characteristics. The synergistic interaction of these shared constituents, particularly the high abundance of esters and phenethyl alcohol, is crucial in defining the quintessential floral, fruity, and mellow sensory profile of honey [20]. Notably, the presence of 2,3-butanediol across all samples points to mild enzymatic or microbial fermentation, a common feature in honey maturation. Ultimately, these common compounds form the chemical basis of the “honey flavor,” upon which the unique volatile signatures of each variety are built, creating their distinct sensory impressions.

#### 3.4.2. Unique Volatile Compounds and Potential Chemical Markers

A comparative analysis of volatile compounds revealed distinct chemical fingerprints for each honey type, enabling their differentiation and botanical origin tracing (Table 5). These unique profiles are characterized by dominant compound classes that define their signature aromas. BCH contained the largest number of characteristic compounds with the most complex chemical composition and a rich multi-layered aroma. Its chromatogram was dominated by esters and terpenoids, which imparted an intense floral–fruity fragrance. The core aroma combination, consisting of phenethyl acetate and (3R,6S)-2,2,6-trimethyl-6-vinyltetrahydro-2H-pyran-3-ol, contributed rose-like, fruity, and woody floral notes. Monoterpenes such as 1,3,8-p-menthatriene added a fresh grassy scent, while C_6_ compounds like (Z)-3-hexen-1-ol acetate and 3-hexenoic acid ethyl ester provided characteristic “green-leaf” top notes. Polycyclic terpenoids such as 2-(4a,8-dimethyl-2,3,4,5,6,7-hexahydro-1H-naphthalen-2-yl) propan-2-ol contributed woody and earthy nuances. Notably, 2,4-di-tert-butylphenol, a known antioxidant compound, was detected and may be related to secondary plant metabolism of the nectar source. Collectively, these compounds formed the distinctive “floral–fruity tone with light woody notes” of BCH.

PPH was characterized by sesquiterpenoids, nitrogen-containing heterocycles (N-heterocycles), and complex terpene ketones, yielding woody-herbal base notes with subtle fruity accents. Cedrol was identified as a major marker compound, imparting a persistent cedarwood scent and serving as a strong indicator of plant origin. N-heterocycles such as pyrazole-4-carboxaldehyde, 1-ethyl-5-methyl- may derive from species-specific secondary metabolism of the nectar plant, providing potential chemical fingerprints. A structurally distinctive terpene ketone, identified as ethanone, 1-(1a,2,3,5,6a,6b-hexahydro-3,3,6a-trimethyloxireno[g]benzofuran-5-yl)-, exhibited high specificity. Despite its low abundance, it shows promise as a unique marker for PPH. This specific volatile profile, which also includes other characteristic compounds, collectively defines the “woody-herbal tone with medicinal nuances” of PPH. This aromatic signature aligns with its traditional therapeutic applications, including “clearing heat, detoxification, and promoting blood circulation”.

RCH displayed a relatively simple yet distinctive volatile profile, characterized by a dominant pattern of fatty acid ethyl esters and aromatic aldehydes, which yields a mellow, sweet, and fruity aroma with subtle almond notes. The main aromatic body and smooth mouthfeel were constituted by medium- to long-chain fatty acid ethyl esters, such as octanoic acid ethyl ester (fruity, wine-like), tetradecanoic acid ethyl ester (waxy, fruity), and tridecanoic acid, 3-hydroxy-, ethyl ester. Although these compounds are common fermentation or aging products in honey, their specific combination and relative abundance serve as discriminative features for RCH. Complementing the esters, aromatic aldehydes such as benzaldehyde, α-ethylidenebenzeneacetaldehyde, and α-ethylidene- contributed nutty and almond-like notes. This characteristic ester-aldehyde synergy not only defines the honey’s “sweet yet slightly bitter” aroma but may also underlie its noted antibacterial potential. Consequently, the marker set of octanoic acid ethyl ester, tetradecanoic acid ethyl ester, and benzaldehyde provides a concise chemical signature for RCH.

In summary, the unique volatile profiles of the three honeys clearly distinguished their botanical sources and dominant aroma types: BCH exhibited a rich floral–fruity aroma, PPH showed a woody-herbal scent, and RCH presented a mellow fruity and nutty fragrance. These differential volatiles can serve as key chemical markers for geographical indication and botanical origin tracing.

#### 3.4.3. Bioactive Potential and Correlation Analysis of Key Volatile Compounds

Literature-based bioactivity profiling of the identified volatiles assigned distinct functional potentials to the three honey varieties. Based on previous studies, BCH volatiles such as 2,4-di-tert-butylphenol and terpene alcohols have been associated with antioxidant activity [31,32], suggesting that BCH may possess strong antioxidant potential. PPH, which is dominated by cedrol and terpene ketones, is inferred to exhibit anti-inflammatory activity [33]. RCH contains phenolic and aldehyde compounds which are reported to enhance antibacterial and anti-inflammatory effects [34]. These associations preliminarily indicate volatile constituents may serve as both sensory markers and chemical indicators of potential bioactivity [32,33]. However, it should be noted that these functional inferences are based on literature and chemical composition alone, and direct experimental confirmation of bioactivity, including bioavailability, synergistic effects with non-volatile components, and the mechanism of action, is required in future studies.

This study provides the first systematic comparison of the volatile profiles of BCH, PPH, and RCH, revealing their distinct chemical fingerprints. These volatile constituents contribute to the diversity of honey’s aroma and reflect the metabolic traits of nectar plants. BCH is characterized by floral–fruity notes rich in oxygenated terpenes; PPH features herbal-woody tones with nitrogenous terpenoids; and RCH centres on ester-aldehyde combinations that produce sweet and nutty scents. These findings emphasise the potential significance of volatile composition in determining the “identity and essence” of honey, offering a scientific basis for origin tracing, quality assessment, and functional studies. Integrating sensory assessment, quantitative volatile profiling and functional assays in future research could help to establish a comprehensive ‘chemical–sensory–bioactivity’ correlation model, guiding the development of high-quality honeys.

## 4. Conclusions

This study systematically compared three *A. cerana* honeys (BCH, PPH and RCH) in terms of their phenolic content, antioxidant capacity, antibacterial activity and volatile profiles. Significant plant-source specificity was observed in both bioactivity and sensory characteristics. BCH had the highest total phenolic content (636.21 ± 17.05 mg GAE/kg) and the strongest antioxidant capacity, as shown by superior DPPH, ABTS and FRAP values (*p* < 0.05). This suggests that phenolic compounds are the main contributors, while non-phenolic compounds may act synergistically. All honeys effectively inhibited *S. aureus* and *E. coli*, with undiluted samples demonstrating the greatest potency. Notably, BCH and PPH surpassed penicillin in their ability to inhibit *S. aureus*, highlighting their potential as natural antibacterial agents. Volatile analysis elucidated the chemical basis for the functional and sensory differences. BCH’s floral–fruity aroma was associated with esters such as geranyl acetate, PPH’s woody/herbal notes with terpenoids such as cedrol, and RCH’s almond-like sweetness with benzaldehyde and fatty acid ethyl esters. These volatiles can be used as reliable markers of botanical origin, and certain compounds (e.g., 2,4-di-tert-butylphenol in BCH) may contribute to bioactivity.

However, it should be noted that all bioactivity data were obtained in vitro and cannot be directly extrapolated to in vivo functionality. The semi-quantitative volatile analysis and the absence of targeted quantification or isolation of non-phenolic actives restrict the interpretation of mechanisms. Future studies should therefore focus on the quantitative analysis of key volatiles and the isolation of non-phenolic bioactives, as well as in vivo or advanced cell-based evaluations, in order to validate biological significance, elucidate synergistic mechanisms and support the development of these honeys as natural functional products. In conclusion, BCH, PPH and RCH are all highly bioactive, with BCH demonstrating the greatest potential for development. The identified volatile markers provide a scientific basis for authenticating the botanical origin of honey and assessing its quality.

## Figures and Tables

**Table 1:** Botanical and geographical origins of the honey samples.

Sample Name	Botanical Origin	Geographical Origin	Collection Time	Color	Average Pollen Percentage (%)	Physical State
PPH	*Polygonum perfoliatum*	Hechi City, Guangxi Zhuang Autonomous Region, China	July 2025	Amber	75.35 ± 5.20	Liquid
RCH	*Rhus chinensis* Mill.		August 2025	Light amber	78.68 ± 3.82	
BCH	*Bauhinia championii* (*Benth.*) *Benth*		September 2025	Dark amber	83.40 ± 6.15	

**Table 2:** EC_50_ values for the scavenging of DPPH free radicals in three types of honey.

Sample Name	Correlation Coefficient (R^2^)	EC_50_ (g/mL)
BCH	0.994	0.015 ± 0.002 ^a^
PPH	0.959	0.185 ± 0.012 ^b^
RCH	0.938	0.207 ± 0.018 ^b^

Different lowercase letters within a column signify significant differences (*p* < 0.05).

**Table 3:** EC_50_ values for the scavenging of ABTS free radicals in three types of honey.

Sample Name	Correlation Coefficient (R^2^)	EC_50_ (g/mL)
BCH	0.9946	0.689 ± 0.118 ^a^
PPH	0.8753	0.720 ± 0.075 ^b^
RCH	0.9988	0.880 ± 0.212 ^c^

Different lowercase letters within a column signify significant differences (*p* < 0.05).

**Table 4:** Diameter of the bacteriostatic zone of honey samples against microorganisms.

Specimen	Inhibition Zone Size (mm)	
	*S. aureus*	*E. coli*
PPH (undiluted)	49.36 ± 0.80 ^a^	30.15 ± 0.42 ^a^
BCH (undiluted)	50.21 ± 0.40 ^a^	18.23 ± 0.14 ^b^
RCH (undiluted)	47.15 ± 0.56 ^b^	27.18 ± 0.50 ^c^
PPH (0.5 g/mL)	38.03 ± 0.80 ^d^	14.02 ^d^
BCH (0.5 g/mL)	37.15 ± 0.70 ^d^	10.14 ± 0.88 ^e^
RCH (0.5 g/mL)	40.23 ± 0.20 ^c^	10.13 ± 0.62 ^e^
Penicillin	28.16 ± 0.44 ^e^	
Streptomycin		36 ± 0.70 ^f^
Water	-	-

Different lowercase letters within a column signify significant differences (*p* < 0.05).

**Table 5:** Core chemical families, unique volatile compounds and potential marker substances in three types of honey.

Honey Type	Core Chemical Families	Potential Key Markers	Inferred Dominant Aroma
BCH	Esters (floral) Terpenoids (mono-, oxygenated) Green-leaf volatiles	Phenethyl acetate (3R,6S)-2,2,6-trimethyl-6-vinyltetrahydro-2H-pyran-3-ol 1,3,8-p-Menthatriene (Z)-3-Hexen-1-ol acetate	Rich floral–fruity with subtle woody and green-leaf notes
PPH	Sesquiterpenes Terpene ketones Nitrogen-containing heterocycles	Cedrol Pyrazole-4-carboxaldehyde, 1-ethyl-5-methyl- 1-(1a,2,3,5,6a,6b-hexahydro-3,3,6a-trimethyloxireno[g]benzofuran-5-yl)-	Woody-herbal tone complemented by medicinal notes
RCH	Fatty acid ethyl esters Aromatic derivatives	Octanoic acid ethyl ester Tetradecanoic acid ethyl ester Benzaldehyd	Mellow fruity-sweet with almond-nutty notes

## Data Availability

The original contributions presented in this study are included in the article/Supplementary Material. Further inquiries can be directed to the corresponding author(s).

## Supplementary Materials

The following supporting information can be downloaded at: https://www.mdpi.com/article/10.3390/foods15010024/s1, Table S1: Volatile Components in *Bauhinia championii* Honey (BCH); Table S2: Volatile Components in *Polygonum perfoliatum* Honey (PPH); Table S3: Volatile Components in *Rhus chinensis* Honey (RCH).

## Abbreviations

The following abbreviations are used in this manuscript:

BCH	*Bauhinia championii* honey
PPH	*Polygonum perfoliatum* honey
RCH	*Rhus chinensis* honey
CFU	colony-forming units

## References

[1] Palma-Morales M., Huertas J.R., Rodríguez-Pérez C.A. (2023). Comprehensive Review of the Effect of Honey on Human Health. Nutrients.

[2] Tlak Gajger I., Dar S.A., Ahmed M.M.M., Aly M.M., Vlainić J. (2025). Antioxidant Capacity and Therapeutic Applications of Honey: Health Benefits, Antimicrobial Activity and Food Processing Roles. Antioxidants.

[3] Rahman M.M., Rahaman M.S., Islam M.R., Rahman F., Mithi F.M., Alqahtani T., Almikhlafi M.A., Alghamdi S.Q., Alruwaili A.S., Hossain M.S. (2021). Role of Phenolic Compounds in Human Disease: Current Knowledge and Future Prospects. Molecules.

[4] Tatipamula V.B., Kukavica B. (2021). Phenolic Compounds as Antidiabetic, Anti-Inflammatory, and Anticancer Agents and Improvement of Their Bioavailability by Liposomes. Cell Biochem. Funct..

[5] Masad R.J., Haneefa S.M., Mohamed Y.A., Al-Sbiei A., Bashir G., Fernandez-Cabezudo M.J., Al-Ramadi B.K. (2021). The Immunomodulatory Effects of Honey and Associated Flavonoids in Cancer. Nutrients.

[6] Guo J., Ding Q., Zhang Z., Zhang Y., He J., Yang Z., Zhou P., Gong X. (2023). Evaluation of the Antioxidant Activities and Phenolic Profile of Shennongjia *Apis cerana* Honey through a Comparison with *Apis mellifera* Honey in China. Molecules.

[7] Schiassi M.C.E.V., de Souza V.R., Lago A.M.T., Carvalho G.R., Curi P.N., Guimarães A.S., Queiroz F. (2021). Quality of Honeys from Different Botanical Origins. J. Food Sci. Technol..

[8] Zhao H., Cheng N., He L., Peng G., Liu Q., Ma T., Cao W. (2018). Hepatoprotective Effects of the Honey of *Apis cerana* Fabricius on Bromobenzene-Induced Liver Damage in Mice. J. Food Sci..

[9] Liu R., Zhang X., Liu H., Huang Y., Zhang Y., Wu Y., Nie J. (2025). Revealing the Key Antioxidant Compounds and Potential Action Mechanisms of *Bauhinina championii* Honey Based on Non-Targeted Metabolomics, Mineralogical Analysis and Physicochemical Characterization. Food Chem..

[10] Liu J., Zeng Y., Sun G., Yu S., Xu Y., He C., Li Z., Jin S., Qin X. (2020). *Polygonum perfoliatum* L., an Excellent Herbal Medicine Widely Used in China: A Review. Front. Pharmacol..

[11] Li H., Lang Y., Liu Z., Song M., Jiang A., Li N., Chen L. (2024). Dynamic Variation in the Aroma Characteristics of Rhus chinensis Honey at Different Stages after Capping. Food Chem..

[12] Costa G., Francisco V., Lopes M.C., Cruz M.T., Batista M.T. (2012). Intracellular Signaling Pathways Modulated by Phenolic Compounds: Application for New Anti-Inflammatory Drugs Discovery. Curr. Med. Chem..

[13] Acevedo F., Torres P., Oomah B.D., de Alencar S.M., Massarioli A.P., Martín-Venegas R., Albarral-Ávila V., Burgos-Díaz C., Ferrer R., Rubilar M. (2017). Volatile and Non-Volatile/Semi-Volatile Compounds and In Vitro Bioactive Properties of Chilean Ulmo (*Eucryphia cordifolia* Cav.) Honey. Food Res. Int..

[14] Kaškonienė V., Venskutonis P.R. (2010). Floral Markers in Honey of Various Botanical and Geographic Origins: A Review. Compr. Rev. Food Sci. Food Saf..

[15] Wu F., Zhao H., Sun J., Guo J., Wu L., Xue X., Cao W. (2021). ICP-MS-Based Ionomics Method for Discriminating the Geographical Origin of Honey of *Apis cerana* Fabricius. Food Chem..

[16] Belay A., Solomon W.K., Bultossa G., Adgaba N., Melaku S. (2015). Botanical origin, colour, granulation, and sensory properties of the Harenna forest honey, Bale, Ethiopia. Food Chem..

[17] Wilczyńska A. (2014). Effect of Filtration on Colour, Antioxidant Activity and Total Phenolics of Honey. LWT-Food Sci. Technol..

[18] Almeida-Muradian L.B., Barth O.M., Dietemann V., Eyer M., Freitas A.S., Martel A.-C., Marcazzan G.L., Marchese C.M., Mucignat-Caretta C., Pascual-Maté A. (2020). Standard Methods for *Apis mellifera* Honey Research. J. Apic. Res..

[19] Balázs V.L., Nagy-Radványi L., Bencsik-Kerekes E., Koloh R., Szabó D., Kocsis B., Kocsis M., Farkas Á. (2023). Antibacterial and Antibiofilm Effect of Unifloral Honeys against Bacteria Isolated from Chronic Wound Infections. Microorganisms.

[20] Zhang C., Zhou S., Wu C., Xu X., Zhu X. (2025). Evaluation of Litchi Honey Quality in Southern China. Foods.

[21] Gośliński M., Nowak D., Kłębukowska L. (2020). Antioxidant Properties and Antimicrobial Activity of Manuka Honey versus Polish Honeys. J. Food Sci. Technol..

[22] Tananaki C., Rodopoulou M.-A., Dimou M., Kanelis D., Liolios V. (2024). The Total Phenolic Content and Antioxidant Activity of Nine Monofloral Honey Types. Appl. Sci..

[23] Hulea A., Obiștioiu D., Cocan I., Alexa E., Negrea M., Neacșu A.G., Hulea C., Pascu C., Costinar L., Iancu I. (2022). Diversity of Monofloral Honey Based on the Antimicrobial and Antioxidant Potential. Antibiotics.

[24] Poulsen-Silva E., Gordillo-Fuenzalida F., Velásquez P., Llancalahuen F.M., Carvajal R., Cabaña-Brunod M., Otero M.C. (2023). Antimicrobial, Antioxidant, and Anti-Inflammatory Properties of Monofloral Honeys from Chile. Antioxidants.

[25] Majewska E., Drużyńska B., Derewiaka D., Ciecierska M., Pakosz P. (2024). Comparison of Antioxidant Properties and Color of Selected Polish Honeys and Manuka Honey. Foods.

[26] Zhang G.Z., Han L.Y., Li S.S., Zheng H.Q., Hu F.L. (2024). Antioxidant and Antimicrobial Properties of Three Monofloral Kinds of Honey from Medicinal Plants. Food Ferment. Ind..

[27] Adgaba N., Al-Ghamdi A., Sharma D., Tadess Y., Alghanem S.M., Ali Khan K., Ansari M.J., Mohamed G.K.A. (2020). Physico-Chemical, Antioxidant and Anti-Microbial Properties of Some Ethiopian Mono-Floral Honeys. Saudi J. Biol. Sci..

[28] Fernández-Estellé M., Hernández-González V., Saurina J., Núñez O., Sentellas S. (2023). Characterization and Classification of Spanish Honeydew and Blossom Honeys Based on Their Antioxidant Capacity. Antioxidants.

[29] Hossain M.L., Lim L.Y., Hammer K., Hettiarachchi D., Locher C. (2023). Determination of Antioxidant and Antibacterial Activities of Honey-Loaded Topical Formulations: A Focus on Western Australian Honeys. Appl. Sci..

[30] Gorlíński M., Nowak D., Pawłowska A., Wojtasiak R., Gasiński A. (2021). Antibacterial Activity of Polish Honeys against Selected Pathogenic Bacteria Including *Staphylococcus aureus* and *Escherichia coli*. LWT-Food Sci. Technol..

[31] Alvarez-Suarez J.M., Gasparrini M., Forbes-Hernandez T.Y., Mazzoni L., Giampieri F. (2014). The Composition and Biological Activity of Honey: A Focus on Manuka Honey. Foods.

[32] Manyi-Loh C.E., Ndip R.N., Clarke A.M. (2011). Volatile compounds in honey: A review on their involvement in aroma, botanical origin determination and potential biomedical activities. Int. J. Mol. Sci..

[33] Koyuncu G. (2025). Determination of volatile compounds of Turkish commercial monofloral honeys by Headspace-solid phase microextraction/gas chromatography-mass spectrometry and sensory evaluation. Int. J. Food Sci. Technol..

[34] Behrouz S., Hosseini M., Rezaee R., Boskabady M.H., Asgharzadeh F., Gholamnezhad Z. (2025). Cedrol ameliorates lipopolysaccharide-induced systemic inflammation and lung injury in rats. Eur. J. Pharmacol..

