# Supplementary material for: Evaluation of the Antioxidant, Antibacterial Activity and Volatile Components of Three Distinctive Apis cerana Honeys
Source: Foods. 2025 Dec 22;15(1):24. doi: 10.3390/foods15010024 (PMC12786090; doi:10.3390/foods15010024)
Supplement: Supplementary file 1 [file foods-15-00024-s001.zip › foods-4008583-supplementary.pdf]

Table S1. Volatile Components in *Bauhinia championii* Honey (BCH)

Number	RT(min)	Chemical Compound	Confidence(%)	Molecular Formula	CAS	Peak Area ($\times 10^6$)
1	2.7112	Dimethyl ether	94.75	C_2H_6O	115-10-6	0.08
2	3.6784	Ethyl Acetate	92.39	$C_4H_8O_2$	141-78-6	53.83
3	4.4121	Butane, 1-methoxy-	82.31	$C_5H_{12}O$	628-28-4	1.99
4	5.7840	Oxetane, 3-(1-methylethyl）-	91.49	$C_6H_{12}O$	10317-17-6	8.7
5	5.8746	1-Butanol, 2-methyl-	83.07	$C_5H_{12}O$	137-32-6	7.97
6	7.3319	2,3-Butanediol, [S-(R*,R*）]-	94.02	$C_4H_{10}O_2$	19132-06-0	0.98
7	8.0991	Propanoic acid, 2-hydroxy-, ethyl ester	94.29	$C_5H_{10}O_3$	97-64-3	0.43
8	9.5043	3-Furanmethanol	90.31	$C_5H_6O_2$	4412-91-3	0.6
9	11.1319	2-Heptanol	94.5	$C_7H_{16}O$	543-49-7	0.81
10	13.8073	1-Heptanol	91.69	$C_7H_{16}O$	111-70-6	0.42
11	15.2265	3-Hexenoic acid, ethyl ester	90.21	$C_8H_{14}O_2$	2396-83-0	0.39
12	15.3097	3-Hexen-1-ol, acetate,（Z）-	92.32	$C_8H_{14}O_2$	3681-71-8	0.36
13	16.3296	1,2-Propanediol, 1-phenyl-	93.91	$C_9H_{12}O_2$	1855-09-0	0.27
14	17.2727	Ethyl dl-2-hydroxycaproate	85.74	$C_8H_{16}O_3$	6946-90-3	0.53

15	17.8580	trans-Linalool oxide（furanoid）	95.71	$C_{10}H_{18}O_2$	34995-77-2	76.67
16	18.8686	Linalool	82.33	$C_{10}H_{18}O$	78-70-6	1.46
17	19.3955	Phenylethyl Alcohol	96.16	$C_8H_{10}O$	1960/12/8	17.74
18	20.0507	1,3,8-p-Menthatriene	91.47	$C_{10}H_{14}$	18368-95-1	0.28
19	20.5689	2,6,6-Trimethyl-2-cyclohexene-1,4-dione	84.72	$C_9H1_2O_2$	1125-21-9	0.2
20	21.4616	1,4-Cyclohexanedione, 2,2,6-trimethyl-	84.09	$C_9H_{14}O_2$	20547-99-3	1.05
21	21.5229	Cyclopentane, butyl-	92.54	C_9H_{18}	2040-95-1	0.78
22	21.6858	(3R,6S）-2,2,6-Trimethyl-6-vinyltetrahydro-2H-pyran-3-ol	87.89	$C_{10}H_{18}O_2$	39028-58-5	0.91
23	21.8790	Butanedioic acid, diethyl ester	96.83	$C_8H_{14}O_4$	123-25-1	5.68
24	21.9790	(3Z,5E）-1,3,5-Undecatriene	83.8	$C_{11}H_{18}$	19883-27-3	0.15
25	22.3418	.alpha.-Terpineol	95.25	$C_{10}H_{18}O$	98-55-5	0.91
26	22.4844	Octanoic acid, ethyl ester	95.13	$C_{10}H_{20}O_2$	106-32-1	3.23
27	22.6949	1,3-Cyclohexadiene-1-carboxaldehyde, 2,6,6-trimethyl-	92.72	$C_{10}H_{14}O$	116-26-7	0.18
28	23.8613	2-Hydroxy-3,5,5-trimethylcyclohex-2-ene-1,4-dione	89.9	$C_9H_{12}O_3$	35692-98-9	0.53
29	24.2473	Benzeneacetic acid, ethyl ester	91.92	$C_{10}H_{12}O_2$	101-97-3	1.19
30	24.4551	(E）-2,6-Dimethylocta-3,7-diene-2,6-diol	80.19	$C_{10}H_{18}O_2$	51276-34-7	0.59
31	24.6771	Acetic acid, 2-phenylethyl ester	97.89	$C_{10}H_{12}O_2$	103-45-7	71.87

32	25.1362	Phenol, 2,3,6-trimethyl-	98.2	$C_9H_{12}O$	2416-94-6	9.03
33	25.3872	1,2,3,4-Tetrahydro-2-naphthol	80.35	$C_{10}H_{12}O$	530-91-6	0.31
34	26.6799	Phenol, 3,4,5-trimethyl-	96.95	$C_9H_{12}O$	527-54-8	1.63
35	27.6309	2,4,6-Cycloheptatrien-1-one, 2-hydroxy-4-(1-methylethyl）-	86.37	$C_{10}H_{12}O_2$	499-44-5	0.99
36	27.9420	Propanoic acid, 2-phenylethyl ester	93.13	$C_{11}H_{14}O_2$	122-70-3	0.66
37	28.9852	Diethyl adipate	86.47	$C_{10}H_{18}O_4$	141-28-6	0.35
38	29.2977	Decanoic acid, ethyl ester	95.7	$C_{12}H_{24}O_2$	110-38-3	3.31
39	33.0545	2,4-Di-tert-butylphenol	90.12	$C_{14}H_{22}O$	96-76-4	0.23
40	35.5878	Ethanone, 1-(1a,2,3,5,6a,6b-hexahydro-3,3,6a-trimethyloxireno[g]benzofuran-5-yl）-	81.56	$C_{13}H_{18}O_3$	80114-25-6	1.03
41	37.3426	2-(4a,8-Dimethyl-2,3,4,5,6,7-hexahydro-1H-naphthalen-2-yl）propan-2-ol	96.06	$C_{15}H_{26}O$	1000411-50-0	0.37

Table S2. Volatile Components in *Polygonum perfoliatum* Honey (PPH)

Number	RT(min)	Chemical Compound	Confidence(%)	Molecular Formula	CAS	Peak Area (×10^{6})
1	2.7187	(2-Aziridinylethyl）amine	85.3	$C_4H_{10}N_2$	4025-37-0	11.87
2	3.6808	Ethyl Acetate	92.11	$C_4H_8O_2$	141-78-6	33.52
3	3.8235	Isobutane	90.87	C_4H_{10}	75-28-5	7.62
4	5.6374	1,3-Dioxolane, 2,4,5-trimethyl-	86.07	$C_6H_{12}O_2$	3299-32-9	1.86
5	5.8761	2-Propenoic acid, ethenyl ester	83.07	$C_5H_6O_2$	2177-18-6	1.1
6	5.8769	1-Butanol, 2-methyl-	89.4	$C_5H_{12}O$	137-32-6	6.9
7	7.3513	2,3-Butanediol, [S-(R*,R*）]-	93.51	$C_4H_{10}O_2$	19132-06-0	0.62
8	8.1018	Propanoic acid, 2-hydroxy-, ethyl ester	95.45	$C_5H_{10}O_3$	97-64-3	0.87
9	11.1238	2-Heptanol	95.97	$C_7H_{16}O$	543-49-7	1.39
10	16.7304	Benzeneacetaldehyde	94.61	C_8H_8O	122-78-1	3.04
11	17.8612	trans-Linalool oxide（furanoid）	95.69	$C_{10}H_{18}O_2$	34995-77-2	95.31
12	18.8703	Linalool	95.78	$C_{10}H_{18}O$	78-70-6	1.94
13	19.0466	1,5,7-Octatrien-3-ol, 3,7-dimethyl-	94.66	$C_{10}H_{16}O$	29957-43-5	3.25

14	19.3961	Phenylethyl Alcohol	96.15	$C_8H_{10}O$	1960/12/8	15.78
15	19.7205	Isophorone	92.72	$C_9H_{14}O$	78-59-1	1.13
16	20.5654	2,6,6-Trimethyl-2-cyclohexene-1,4-dione	94.18	$C_9H_{12}O_2$	1125-21-9	1.11
17	21.5843	Benzoic acid, ethyl ester	96.56	$C_9H_{10}O_2$	93-89-0	1.27
18	21.8857	Butanedioic acid, diethyl ester	96.86	$C_8H_{14}O_4$	123-25-1	5.37
19	22.7053	1,3-Cyclohexadiene-1-carboxaldehyde, 2,6,6-trimethyl-	80.75	$C_{10}H_{14}O$	116-26-7	0.27
20	23.4300	4-Methyleneisophorone	94.28	$C_{10}H_{14}O$	20548-00-9	0.29
21	23.8693	2-Hydroxy-3,5,5-trimethylcyclohex-2-ene-1,4-dione	88.15	$C_9H_{12}O_3$	35692-98-9	0.44
22	24.2489	Benzeneacetic acid, ethyl ester	91.8	$C_{10}H_{12}O_2$	101-97-3	3.44
23	24.6777	Acetic acid, 2-phenylethyl ester	97.06	$C_{10}H_{12}O_2$	103-45-7	2.04
24	25.1371	Phenol, 2,3,6-trimethyl-	98.17	$C_9H_{12}O$	2416-94-6	9.01
25	25.9848	Nonanoic acid, ethyl ester	85.23	$C_{11}H_{22}O_2$	123-29-5	0.7
26	26.6870	Phenol, 3,4,5-trimethyl-	97.62	$C_9H_{12}O$	527-54-8	67.78
27	27.1675	Hexanoic acid, 3-hydroxy-, ethyl ester	84.17	$C_8H_{16}O_3$	2305-25-1	3.93
28	27.6293	2,4,6-Cycloheptatrien-1-one, 2-hydroxy-4-(1-methylethyl）-	86.84	$C_{10}H_{12}O_2$	499-44-5	5.09
29	29.0952	2-Buten-1-one, 1-(2,6,6-trimethyl-1,3-cyclohexadien-1-yl）-,（E）-	93.72	$C_{13}H_{18}O$	23726-93-4	0.4
30	35.2741	Pyrazole-4-carboxaldehyde, 1-ethyl-5-methyl-	81.02	$C_7H_{10}N_2O$	1000273-81-2	1.28

31	35.5856	Ethanone, 1-(1a,2,3,5,6a,6b-hexahydro-3,3,6a-trimethyloxireno[g]benzofuran-5-yl）-	83.43	$C_{13}H_{18}O_3$	80114-25-6	2.97
32	36.0052	Dodecanoic acid, ethyl ester	93.72	$C_{14}H_{28}O_2$	106-33-2	1.17
33	36.5797	Cedrol	93.04	$C_{15}H_{26}O$	77-53-2	0.43
34	40.5151	Tetradecanoic acid, ethyl ester	94.95	$C_{16}H_{32}O_2$	124-06-1	2.31
35	43.3229	Hexadecanoic acid, ethyl ester	94.11	$C_{18}H_{36}O_2$	628-97-7	2.68

Table S3 Volatile Components in *Rhus chinensis* Honey (RCH)

Number	RT(min)	Chemical Compound	Confidence(%)	Molecular Formula	CAS	Peak Area ($\times10^6$)
1	3.6741	Ethyl Acetate	47.11	$C_4H_8O_2$	141-78-6	92.36
2	3.8168	Isobutane	8.93	C_4H_{10}	75-28-5	90.74
3	5.7868	Oxetane, 3-(1-methylethyl）-	5.22	$C_6H_{12}O$	10317-17-6	92.25
4	5.8734	1-Butanol, 2-methyl-	7.71	$C_5H_{12}O$	137-32-6	85.73
5	6.6898	1,3-Dioxolane, 2,4,5-trimethyl-	0.58	$C_6H_{12}O_2$	3299-32-9	90.54
6	7.3485	2,3-Butanediol, [S-(R*,R*）]-	0.69	$C_4H_{10}O_2$	19132-06-0	95.42
7	8.0862	Propanoic acid, 2-hydroxy-, ethyl ester	0.58	$C_5H_{10}O_3$	97-64-3	96.29
8	11.1086	2-Heptanol	1.04	$C_7H_{16}O$	543-49-7	92.58
9	13.4609	Benzaldehyde	0.63	C_7H_6O	100-52-7	96.33
10	16.7202	Benzeneacetaldehyde	10.33	C_8H_8O	122-78-1	95.49
11	18.4586	trans-Linalool oxide（furanoid）	20.38	$C_{10}H_{18}O_2$	34995-77-2	95.44
12	18.8611	Linalool	1.32	$C_{10}H_{18}O$	78-70-6	92.78
13	19.0370	1,5,7-Octatrien-3-ol, 3,7-dimethyl-	4.22	$C_{10}H_{16}O$	29957-43-5	94.89
14	19.3914	Phenylethyl Alcohol	19.57	$C_8H_{10}O$	1960/12/8	96.61
15	19.7063	Isophorone	2.48	$C_9H_{14}O$	78-59-1	95.53

16	20.5553	2,6,6-Trimethyl-2-cyclohexene-1,4-dione	2.85	$C_9H_{12}O_2$	1125-21-9	97.31
17	21.5710	Benzoic acid, ethyl ester	1.75	$C_9H_{10}O_2$	93-89-0	96.85
18	21.8760	Butanedioic acid, diethyl ester	5.97	$C_8H_{14}O_4$	123-25-1	97.02
19	22.3296	.alpha.-Terpineol	0.66	$C_{10}H_{18}O$	98-55-5	93.64
20	22.4805	Octanoic acid, ethyl ester	0.3	$C_{10}H_{20}O_2$	116-26-7	88.78
21	22.6925	1,3-Cyclohexadiene-1-carboxaldehyde, 2,6,6-trimethyl-	0.34	$C_{10}H_{14}O$	20548-00-9	93.73
22	23.4122	4-Methyleneisophorone	0.1	$C_{10}H_{14}O$	35692-98-9	90.86
23	23.8519	2-Hydroxy-3,5,5-trimethylcyclohex-2-ene-1,4-dione	2.33	$C_9H_{12}O_3$	101-97-3	92.62
24	24.2379	Benzeneacetic acid, ethyl ester	0.67	$C_{10}H_{12}O_2$	103-45-7	97.12
25	24.6728	Acetic acid, 2-phenylethyl ester	9.73	$C_{10}H_{12}O_2$	2416-94-6	98.22
26	25.1303	Phenol, 2,3,6-trimethyl-	0.57	$C_9H_{12}O$	4411-89-6	95.49
27	25.2694	Benzeneacetaldehyde, .alpha.-ethylidene-	0.81	$C_{10}H_{10}O$	123-29-5	92.02
28	25.9725	Nonanoic acid, ethyl ester	90.05	$C_{11}H_{22}O_2$	527-54-8	97.6
29	26.6808	Phenol, 3,4,5-trimethyl-	4.18	$C_9H_{12}O$	2305-25-1	83.86
30	27.1612	Hexanoic acid, 3-hydroxy-, ethyl ester	6.5	$C_8H_{16}O_3$	499-44-5	86.43
31	27.6231	2,4,6-Cycloheptatrien-1-one, 2-hydroxy-4-(1-methylethyl）-	0.26	$C_{10}H_{12}O_2$	23726-93-4	93.82
32	29.0892	2-Buten-1-one, 1-(2,6,6-trimethyl-1,3-cyclohexadien-1-yl）-,（E）-	0.61	$C_{13}H_{18}O$	110-38-3	93.86

33	29.2870	Decanoic acid, ethyl ester	0.61	$C_{12}H_{24}O_2$	107141-15-1	84.95
34	33.8031	Tridecanoic acid, 3-hydroxy-, ethyl ester	0.53	$C_{15}H_{30}O_3$	106-33-2	92.9
35	35.9943	Dodecanoic acid, ethyl ester	0.73	$C_{14}H_{28}O_2$	124-06-1	93.69
36	40.5117	Tetradecanoic acid, ethyl ester	0.14	$C_{16}H_{32}O_2$	84-69-5	95.36
37	41.7515	1,2-Benzenedicarboxylic acid, bis(2-methylpropyl） ester	1.09	$C_{16}H_{22}O_4$	628-97-7	93.28
38	43.3202	Hexadecanoic acid, ethyl ester	47.11	$C_{18}H_{36}O_2$	141-78-6	92.36

6
